# N-glycosylation proteome enrichment analysis in kidney reveals differences between diabetic mouse models

**DOI:** 10.1186/s12014-016-9123-z

**Published:** 2016-10-15

**Authors:** Leena Liljedahl, Maiken Højgaard Pedersen, Jenny Norlin, James N. McGuire, Peter James

**Affiliations:** 1 Department of Immunotechnology, Lund University, House 406, Medicon Village, 221 83 Lund, Sweden; 2 Novo Nordisk A/S, Novo Nordisk Park, 2760 Måløv, Denmark

**Keywords:** N-linked glycosylation, N-glycosylation, Diabetic nephropathy, Proximal tubules, Glomerulus, Mass spectrometry, Insulin, STZ, db/db

## Abstract

**Background:**

Diabetic nephropathy (DN) is a late complication in both type 1 diabetes mellitus (T1DM) and T2DM. Already at an early stage of DN morphological changes occur at the cell surface and in the extracellular matrix where the majority of the proteins carry N-linked glycosylations. These glycosylated proteins are highly important in cell adhesion and cell–matrix processes but not much is known about how they change in DN or whether the distinct etiology of T1DM and T2DM could have an effect on their abundances.

**Method:**

We enriched for the N-glycosylated kidney proteome in db/db mice dosed with insulin or vehicle, in streptozotocin-induced (STZ) diabetic mice and healthy control mice dosed with vehicle. Glycopeptides were analyzed with label-free shotgun mass spectrometry and differential protein abundances identified in both mouse models were compared using multivariate analyses.

**Results:**

The majority of the N-glycosylated proteins were similarly regulated in both mouse models. However, distinct differences between the two mouse models were for example seen for integrin-β1, a protein expressed mainly in the glomeruli which abundance was increased in the STZ diabetic mice while decreased in the db/db mice and for the sodium/glucose cotransporter-1, mainly expressed in the proximal tubules which abundance was increased in the db/db mice but decreased in the STZ diabetic mice. Insulin had an effect on the level of both glomerular and tubular proteins in the db/db mice. It decreased the abundance of G-protein coupled receptor-116 and of tyrosine-protein phosphatase non-receptor type substrate-1 away from the level in the healthy control mice.

**Conclusions:**

Our finding of differences in the N-glycosylation protein profiles in the db/db and STZ mouse models suggest that the etiology of DN could give rise to variations in the cell adhesion and cell–matrix composition in T1DM and T2DM. Thus, N-glycosylated protein differences could be a clue to dissimilarities in T1DM and T2DM at later stages of DN. Furthermore, we observed insulin specific regulation of N-glycosylated proteins both in the direction of and away from the abundances in healthy control mice.

**Electronic supplementary material:**

The online version of this article (doi:10.1186/s12014-016-9123-z) contains supplementary material, which is available to authorized users.

## Background

The prevalence of both T1DM and T2DM is increasing and together they affect over 340 million people worldwide. The insulin-dependent T1DM covers 5–10 % of the cases where as T2DM comprise 90 % of the disease and the prevalence increases more rapidly [1–3]. Despite different etiology, both T1DM and T2DM result in a 20–30 % occurrence of DN. Type 2 DM patients often go undiagnosed for several years, which means that DN often is diagnosed at a later stage in development. The larger heterogeneity of T2DM patients is reflected in larger structural differences in the kidneys of patients with DN compared to T1DM patients with DN [4, 5]. The structural changes of T1DM and T2DM during progression of DN are however roughly similar [5, 6] with tubular and glomerular basement membrane (BM) thickening, extracellular matrix (ECM) and mesangial expansion and changes in the proteoglycan structure both at the cell surface and in the extracellular space [7].

In the kidney-specific processes of blood filtering, protein retention and reabsorption of fluid and small molecules, the extracellular matrix and its connection to cellular surfaces are regions of high functional importance. In the glomeruli the podocytes guard the slit diaphragm and prevent the loss of larger molecules from the blood to the primary urine and in the tubules solute and water reabsorption is managed [8]. A large proportion of the extracellular and cell surface proteins involved in those processes, including proteoglycans, carry glycosylation on asparagine (N) residues [9]. The N-linked glycosylation pattern is controlled by enzymatic processes as compared to the non-enzymatic, unspecific glucose dependent glycation of proteins, measured by the level of glycated hemoglobin-A1c (HbA1c%) [10, 11].

Addressing changes in the extracellular space and the cell surface can be a challenge since this sub-proteome may be difficult to map due to technical aspects in a regular proteome analysis or in a non-biased way in antibody based analysis [12]. Changes in the N-glycosylated proteome have repeatedly been shown in various diseases and cell-types [12–15]. Since N-linked glycosylation is a hallmark of cell membrane and extracellular proteins [16, 17] and early kidney changes in DN are observed at those locations [8] we enriched for glycosylated peptides with hydrazide capture [18] to target this sub proteome. By using label free shotgun mass spectrometry, we compared the N-glycosylated fraction of the kidney proteomes from two regularly used diabetic mouse models; the STZ-induced diabetic mice with reduced pancreatic beta cell mass and the obese db/db mice with deficient leptin receptor signaling leading to obesity, insulin resistance and finally overt diabetes. Both mouse models are characterized by having early signs of DN in the form of increased albumin excretion rates (AER) [19] driven by hyperglycemia.

Hyperglycemia has been shown to promote DN by increasing oxidative stress [20, 21] and maintaining stable normal blood glucose (BG) levels has a paramount position in diabetes care [22, 23]. In T1DM, insulin is the only treatment option adequately lowering BG levels. Type 2 DM can be managed with several different compounds in the initial phases in order to rescue the reduced insulin sensitivity. At later stages in T2DM insulin administration can be required due to decreased beta-cell mass and impaired insulin secretion [24]. The effects of insulin on the N-glycosylation pattern in DN are not known but changes in proteoglycans are reported in DN and altered glycosylation levels are well known features from diseases like cancer [13]. In the kidneys of STZ diabetic rats, the glycosylation pattern was recently shown to be altered over time [25], however, it is not known if the different etiologies of diabetes give rise to variations in the glycosylation pattern in DN.

We sought to compare the N-glycosylated protein profiles in the kidneys of the db/db and STZ induced diabetic mouse models since the two models represent separate etiologies of diabetes and both exhibit traits of early stage DN [19]. In addition, the effects of insulin on N-glycosylation was investigated in the db/db mouse model kidney to elucidate its influence on the extracellular and cell surface proteome as previously described by Bausch-Fluck et al. [15]. Insulin has earlier been reported to contribute to podocyte survival which is important for maintaining proper glomerular function [26, 27].

## Results

### Experimental design and mouse parameters

**Fig. 1:**
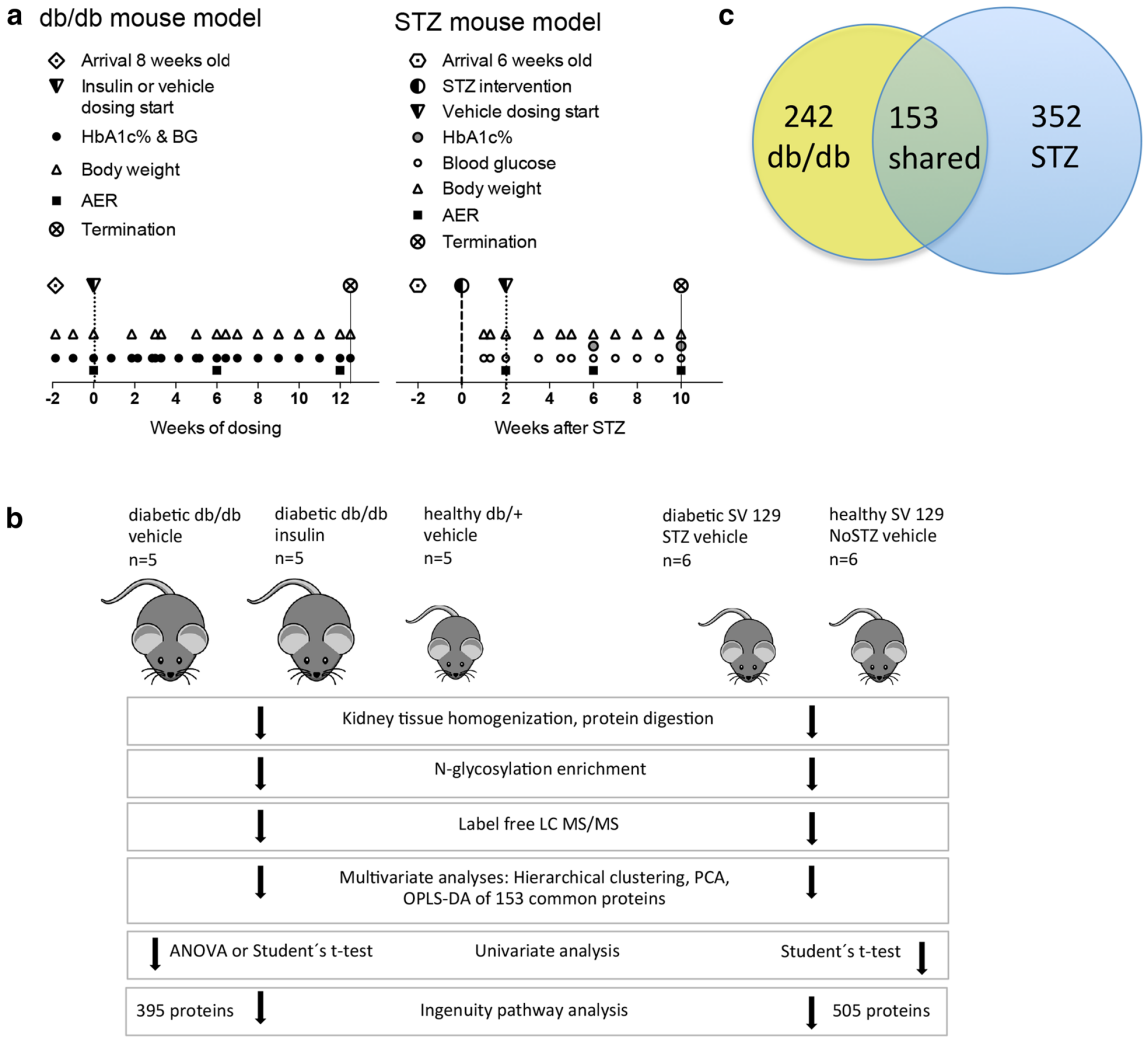
Timeline, workflow and result overview for the db/db and STZ mouse models. **a** The timeline for the db/db and STZ mouse models with indications for time of arrival, STZ intervention, dosing start of insulin or vehicle, analyses, measurements and termination. Blood glucose and body weight were monitored weekly in both mouse models, HbA1c% was analyzed weekly in the db/db model and 6 and 10 weeks after STZ intervention in the STZ mouse model. The albumin excretion rate, AER, was analyzed at three time points in both mouse models; in the db/db model at baseline, 6 and 12 weeks after insulin dosing start and in the STZ model at baseline, 6 and 10 weeks. The STZ study was terminated 10 weeks after the STZ intervention (animals 18 weeks old) and the db/db study was terminated 12.5 weeks after baseline (dosing start of insulin or vehicle) when the animals were 22 weeks old. In both mouse models the included diabetic animals had elevated AER and BG over 16 mM. **b** The workflow for protein purification, data and statistical analyses for the db/db and STZ mouse models. In **c** a Venn diagram of the N-glycosylated proteins in the db/db and STZ mouse models is shown. The protein quantification is based on N-glycosylated unique peptides. In total, 395 N-glycosylated proteins were identified in the db/db mouse model and 505 were identified in the STZ mouse model with *P* < 0.05. All 395 and 505 proteins were respectively used in the IPA as the final step shown in panel B. 153 proteins were identified in both mouse models and those proteins were used in the multivariate analyses

This work uses N-glycosylation capture to compare the N-glycosylated kidney proteomes of the STZ induced diabetic and db/db mouse models, two mouse models frequently used in diabetes research. Both mouse models reflect the early stage of DN and do not progress to develop the severe types of DN and end stage renal disease (ESRD) seen in human diabetic subjects due to their shorter life span. A schematic timeline for the two mouse models is illustrated in Fig. 1a and the proteomics workflow is shown in Fig. 1b.

The STZ diabetic mice reached 33.4 ± 1.45 mM in BG 10 weeks after diabetes induction relative to a fairly stable level of 6.06 ± 1.28 mM throughout the study in the healthy NoSTZ control group. Body weight in the STZ mice did not change much from baseline and was ~5 g lower than controls at termination (25.3 ± 1.2 vs. 30 ± 0.5 g).

The AER was ~tenfold higher in the STZ mice at 578 ± 117 µg/24 h compared with 55.7 ± 12.0 µg/24 h in the NoSTZ controls at the final sampling time point.

The db/db mice did not reach the same level of hyperglycemia, but peaked at 23.12 ± 2.54 mM relative to 8.91 ± 0.28 mM in the db/+ controls at 22 weeks of age. In spite of the lower levels of glycaemia in the db/db mice, HbA1c% was similar or slightly higher at termination compared to the STZ mice (8.52 ± 0.7 % for the db/db and 7.60 ± 0.13 % for the STZ mice), indicating that the accumulated diabetic burden was comparable in the two mouse models. Diabetes in the db/db model comes secondary to overeating and body weight in this model peaked at 54 ± 3.2 versus 31 ± 0.8 g at 16 weeks of age (db/db vs. db/+). After this the body weight stabilized indicating that insulin production in the mice failed to keep up with the increasing insulin resistance the animals develop [19]. The AER was increased about eightfold in the db/db model (930.2 ± 184.8 µg/24 h in db/db vs. 112.2 ± 54.5 µg/24 h in db/+), however, both the healthy db/+ controls and diabetic db/db mice had about twice the AER as the SV129 mice indicating an effect of the background. In the db/db insulin mice HbA1c% was slightly increased at baseline compared to the db/db vehicle mice but after 12 weeks of insulin dosing, at the age of 22 weeks, HbA1c% was reduced in the db/db insulin mice (6.78 ± 0.23 %) compared to the db/db vehicle mice (8.52 ± 0.7 %). At 22 weeks of age there was no significant difference in AER or BG, but the body weight continued to increase in the db/db insulin mice and was after 12 weeks of dosing 62.3 ± 0.9 g compared to 53.7 ± 3.8 g in the db/db vehicle mice. Mouse parameters can be seen in Table 1.

**Table 1:** Mouse parameters in the db/db and STZ mouse models

	db/db mouse model				STZ mouse model		
	Control db/+ (n = 5)	db/db (n = 5)	Insulin db/db (n = 5)	*P* value^A^	Control (STZ) (n = 6)	STZ (n = 6)	*P* value^B^
	*10* *weeks old, baseline (insulin dosing start)*				*10* *weeks old, baseline, 2* *weeks after STZ*		
BG (mM)	8.85 ± 0.48	19.27 ± 2.29	24.34 ± 1.28	<0.002^§^ <0.001^$^	6.50 ± 0.27	22.1 ± 1.60	<0.001
HbA1c (%)	4.06 ± 0.08	6.44 ± 0.48	7.88 ± 0.44	<0.003^§^ <0.001^$^ <0.05^€^	–	–	NA
AER (μg/24 h)	42.5 ± 9.8, n = 4	420.8 ± 111.9	529.3 ± 115	<0.017^§^ <0.06^$^	40.8 ± 16.2, n = 5	408 ± 114	<0.033
BW (g)	27.1 ± 0.9	46.66 ± 0.5	47.92 ± 0.2	<0.001^§,$^	25.2 ± 1.1	23.6 ± 0.4	ns
	*16* *weeks old, 6* *weeks after insulin dosing start*				*14* *weeks old, 6* *weeks after STZ*		
BG (mM)	8.4 ± 0.33	22.88 ± 1.86	16.14 ± 1.61	<0.001^§^ <0.007^$^ <0.017^€^	5.63 ± 0.25	30.9 ± 3.19	<0.001
HbA1c (%)	4.16 ± 0.10	7.94 ± 0.62	7.32 ± 0.23	<0.001^§,$^	3.62 ± 0.031	6.95 ± 0.1	<0.001
AER (μg/24 h)	59.4 ± 13.3, n = 4	855.3 ± 272	488.6 ± 61.2	<0.024^§^	55.6 ± 5.39, n = 5	424 ± 71	<0.012
BW (g)	31 ± 0.8	54 ± 3.2	57.2 ± 1.1	<0.001^§,$^	28.1 ± 1.0	24.8 ± 0.5	<0.018
	*22* *weeks old, 12* *weeks after insulin dosing start*				*18* *weeks old, 10* *weeks after STZ*		
BG (mM)	8.91 ± 0.28	23.12 ± 2.54	21.23 ± 1.49	<0.001^§,$^	8.57 ± 1.21	33.4 ± 1.45	<0.001
HbA1c (%)	4.3 ± 0.07	8.52 ± 0.70	6.78 ± 0.23	<0.001^§^ <0.005^$^ <0.035^€^	3.75 ± 0.06	7.60 ± 0.13	<0.001
AER (μg/24 h)	112.2 ± 54.5, n = 4	930.2 ± 184.8	795.1 ± 183.8	<0.015^§^ <0.039^$^	55.7 ± 12.0, n = 5	578 ± 117	<0.004
BW (g)	32.7 ± 0.7	53.7 ± 3.8	62.3 ± 0.9	<0.001^§,$^ <0.05^€^	30 ± 0.5	25.3 ± 1.2	<0.007

The obtained mouse parameters in the db/db and STZ mouse models show that the db/db vehicle, db/db insulin and STZ mice are diabetic and display a similar degree of diabetes and DN. The data shown at baseline correspond to dosing start of vehicle or insulin in both models (2 weeks after STZ intervention). For comparisons within the db/db mouse model one-way ANOVA with Tukey post hoc test was used^A^. The *P* values for the pairwise comparison within the db/db mouse model is shown as § (db/+ vs. db/db vehicle), $ (db/+ vs. db/db insulin) and € (db/db vehicle vs. db/db insulin). For the STZ mouse model two-tailed Student’s t test for equal variance was used^B^ for *P* value calculations. Data are presented as mean ± SEM, *P* < 0.05 is considered significant

### Visualizing the STZ and db/db mouse models with multivariate analyses and clustering

**Fig. 2:**
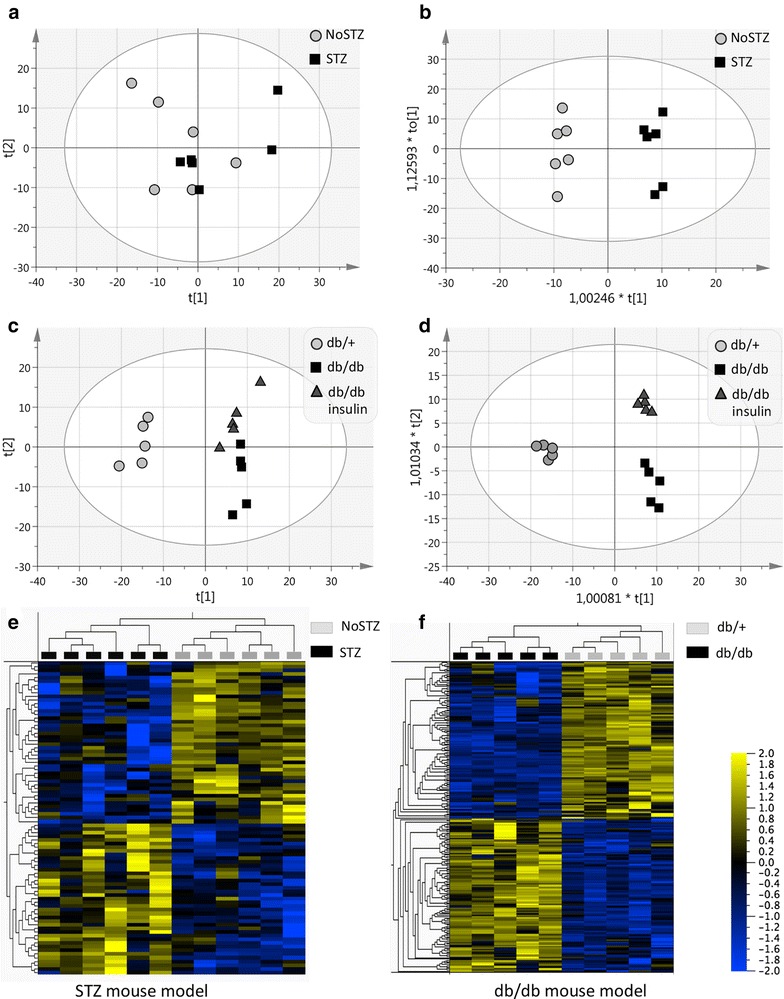
Multivariate analyses. **a** A PCA and **b** an OPLS-DA of the STZ mouse model where all 505 identified proteins were included. The healthy NoSTZ and diabetic STZ mouse groups were not well separated in the unsupervised PCA, but were well separated in the supervised OPLS-DA. **c** A PCA and **d** an OPLS-DA of the db/db mouse model including all 395 identified proteins. The healthy db/+ group was clearly separated from the db/db vehicle and db/db insulin groups in both multivariate models. In the PCA, the two db/db groups were not well separated, but they were clearly separated in the OPLS-DA. Statistics of the multivariate models are shown in an Additional file 2: Panel A. **e** A heat map with hierarchical clustering of the NoSTZ and STZ mouse groups including 87 proteins with *P* < 0.05 cutoff. Panel F shows a heat map with hierarchical clustering of the db/+ and db/db mouse groups including 227 proteins with *P* < 0.05 cutoff. The clustering was done using Qlucore v. 3.2

Analyses of the N-glycosylated sub-proteome does not yield as many identified proteins as regular proteomics analyses do, but provides the opportunity of examining otherwise undetected proteins [12]. A challenge is that the proteins need to include a unique peptide with the NXS/T N-glycosylation motif. Approximately 50 % of the identified peptides in both the STZ and db/db datasets carried an Asn to Asp modification. Protein assembly based on the formerly N-glycosylated peptides resulted in 505 identified proteins from 2602 formerly N-glycosylated peptides in the STZ mouse model (see Additional file 1a) and 395 proteins from 3564 highly formerly N-glycosylated peptides in the db/db mouse model (see Additional file 1b). Of the identified proteins in the two mouse models, 153 proteins were identified as shared in both as illustrated in Fig. 1c. The fact that several proteins only were identified in one mouse model does not mean that the protein not is present in the other mouse model. It is most likely a result of under-sampling or low protein abundance resulting in no unique detected formerly N-glycosylated peptide in those proteins. All the proteins within each mouse model were included in the principal component analysis (PCA) and orthogonal partial least square discriminant analysis (OPLS-DA), the statistics are shown in an Additional file 2: Panel A. In the PCA of the STZ mouse model, the STZ and NoSTZ groups were not clearly separated (Fig. 2a). A 3D model shown in an Additional file 2: Panel B revealed though that there was a degree of separation but the first and second principal components were not separating the groups. The separation of the groups in the PCA of the STZ mice seemed to be more dependent on the degree of perfusion of the kidneys. The supervised OPLS-DA clearly separated the STZ and NoSTZ mouse groups (Fig. 2b). The healthy db/+ mice were clearly separated from the two db/db mouse groups dosed with insulin or vehicle in the PCA (Fig. 2c). Here there was a weak separation of the db/db insulin and db/db vehicle groups, a turned 3D plot is shown in an Additional file 2: Panel C. In the OPLS-DA the db/db vehicle and insulin groups were clearly separated (Fig. 2d). Applying hierarchical clustering on the STZ mouse model resulted in 87 proteins with *P* < 0.05 and there was a clear separation between the expression pattern in the healthy NoSTZ and the diabetic STZ mice (Fig. 2e). The hierarchical clustering of the healthy db/+ and db/db vehicle groups resulted in 227 proteins with *P* < 0.05 (Fig. 2f). The 153 proteins that were identified in both mouse models were selected in the respective OPLS analysis of the two mouse models. The proteins not identified in both datasets were excluded in the continued comparison of the N-glycosylated proteome. The reason for only including proteins identified in both mouse models, is that absent proteins in one mouse model in fact most likely still could be present in the mouse model without being detected in the MS analysis due to low abundance resulting in no unique identified formerly N-glycosylated peptides. Examples of a few of the proteins only identified in the STZ mouse model can be seen in an Additional file 3: Panels A–B. In the OPLS analyses, proteins with a SIMCA variable of importance for the projection (VIP) score above 1 in both mouse models were selected, resulting in 27 shared proteins of importance in the separation of the healthy and diabetic mice in both the STZ and the db/db mouse model. These proteins were examined with univariate analyses.

### Quantitative comparison of the N-glycosylated proteomes of the mouse models

**Fig. 3:**
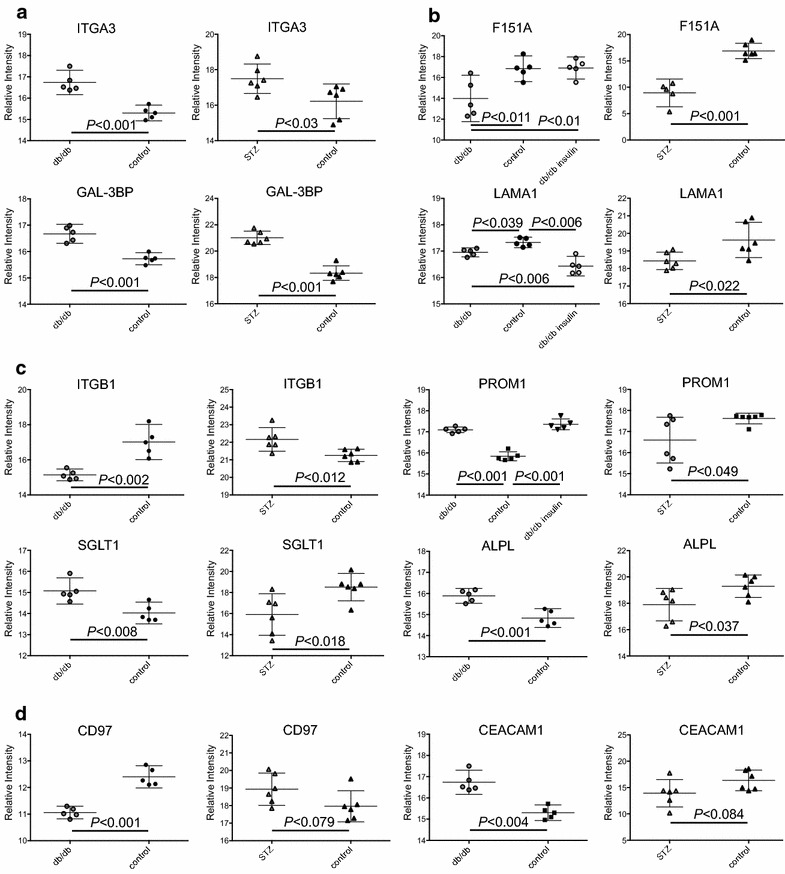
Differences and similarities between the db/db and STZ mouse model. Panel A shows proteins with higher protein abundance in the STZ and db/db vehicle mouse groups compared to the healthy NoSTZ and db/+ control groups in the two mouse models. Panel B shows proteins with lower abundances in the STZ and db/db vehicle mouse groups compared to their healthy littermates. Panel C shows proteins with differently affected protein abundances in the two mouse models. Panel D shows proteins with significantly regulated protein abundance in the db/db mouse model and a trend to significant regulation in the STZ mouse model and thereby a trend towards differently affected protein regulation in the two mouse models. Two-tailed Students t test was used for calculation of significance level when 2 groups were included and one-way ANOVA with Tukey post hoc test was used for the calculations including the db/db insulin group in the db/db mouse model. Graphs are shown with mean and 95 % CI and *P* < 0.05 is considered significant

The univariate analyses revealed that 11 of the 27 shared proteins of importance had significantly changed protein abundances when compared between the diabetic and the healthy mice in both mouse models (Table 2). Mean ± SD, numbers of peptides used in quantitation and Mascot score for the 27 proteins can be seen in an Additional file 1c. The abundances of the three proteins galectin-3 binding-protein (GAL-3BP), integrin-α3 (ITGA3) and lysosomal membrane glycoprotein-1 (LAMP1) were increased in the diabetic mice in both models and the four proteins family with sequence similarity 151, member-A (F151A), cadherin-related family member 5 (CDHR5), meprin-α subunit-β (MEP1B) and high-affinity aspartate/glutamate transporter-6 (SLC1A6) were decreased in the diabetic mice compared to the healthy mice in both mouse models, examples are shown in Fig. 3a, b. An inversed protein regulation in the STZ and db/db mice was seen for the four proteins ITGB1, SGLT1, prominin-1 (PROM1) and alkaline phosphatase (ALPL) as seen in Fig. 3c. For the remaining 16 of the 27 common most important proteins the univariate analyses revealed significant change in the protein abundance in the db/db mouse model but not in the STZ mouse model. The 16 proteins are listed in an Additional file 1d. Among those 16 proteins were the sodium/potassium-transporting ATPase subunit-β1 (ATP1B1) and integrin-αM (ITGAM). Although no significant change was seen in the univariate analysis, the 16 proteins were important in the multivariate modeling of the STZ and healthy NoSTZ mouse groups. There was a trend towards changed protein abundances in three of the proteins and in two of them, the adhesion G protein-coupled receptor E5 (CD97) and carcinoembryonic antigen-related cell adhesion molecule-1a (CEACAM1), the trend was towards an opposite protein regulation in the STZ mouse model compared to the db/db mouse model (Fig. 3d) whereas the serine/cysteine peptidase inhibitor, clade A6 (SERPINA6) had a trend towards abundances similar to the ones in the db/db mouse model.

**Table 2:** Selected proteins identified in both mouse models with opposite or similar protein regulation

Uniprot ID	Description	Short name	STZ versus NoSTZ		db/db versus db/+ db/db insulin versus dbdb^B^		Kidney Compartment^C^
			Protein abuncance level	*P* value	Protein abundance level	*P* value	
P09055	Integrin beta-1	ITGB1	Up	<0.012	Down	<0.002	G = H, T = L
Q8C3K6	Sodium/glucose cotransporter 1	SGLT1	Down	<0.018	Up	<0.008	T = M
O54990^A^	Prominin-1	PROM1	Down	<0.049	Up	<0.001	T = H
Q3TQ02	Alkaline phosphatase, unreviewed	ALPL	Down	<0.037	Up	<0.001	T = M
Q3TA96	Lysosomal membrane glycoprotein 1, unreviewed	LAMP1	Up	<0.011	Up	<0.018	G = H, T = H
Q07797	Galectin-3-binding protein	GAL-3BP	Up	<0.001	Up	<0.001	G = L, T = L
Q62470-2	Integrin alpha-3, isoform 2	ITGA3	Up	<0.03	Up	<0.001	G = M, T = H
Q8QZW3	Family with sequence similarity 151, member A	F151A	Down	<0.001	Down Up^B^	<0.011 <0.01^B^	T = H
A0PJK7	Cadherin-related family member 5, unreviewed	CDHR5	Down	<0.016	Down Tr up^B^	<0.006 <0.099^B^	T = H
Q61847-2	Meprin A subunit beta, isoform 2	MEP1B	Down	<0.008	Down	<0.002	ND, RNA ND
Q6IR20	Solute carrier organic anion transporter family, member 1a6, unreviewed	SLC1A6	Down	<0.030	Down Up^B^	<0.001 <0.032^B^	G = L, T = L

11 of the 27 VIP proteins had significantly different abundances in both the STZ and the db/db mouse models. Uniprot accession number, name of the protein and a short name is shown for all included proteins together with the direction of change in protein abundance level in the diabetic mice compared to the healthy mice within each mouse model and the *P* value. ^A^In PROM1, there was significant difference in intragroup variance. Two-tailed Student’s t test for equal variance was used for *P* value calculations except for the cases where insulin had an effect on the protein level, indicated by^B^. One-way ANOVA with Tukey post hoc test for correction of multiple comparisons was used for db/db insulin and db/db vehicle comparisons. *P* < 0.05 was considered significant. ^C^Kidney compartments were obtained from The Human Protein Atlas [28] using antibody staining where *G* glomerulus, *T* tubulus, *ND* no detected protein, *L* low, *M* medium, *H* high protein expression, RNR means that RNR was detected in the indicated compartment but no protein expression has been confirmed. *Tr* trend, with *P* < 0.1, > 0.05

### Ingenuity pathway analysis

**Fig. 4:**
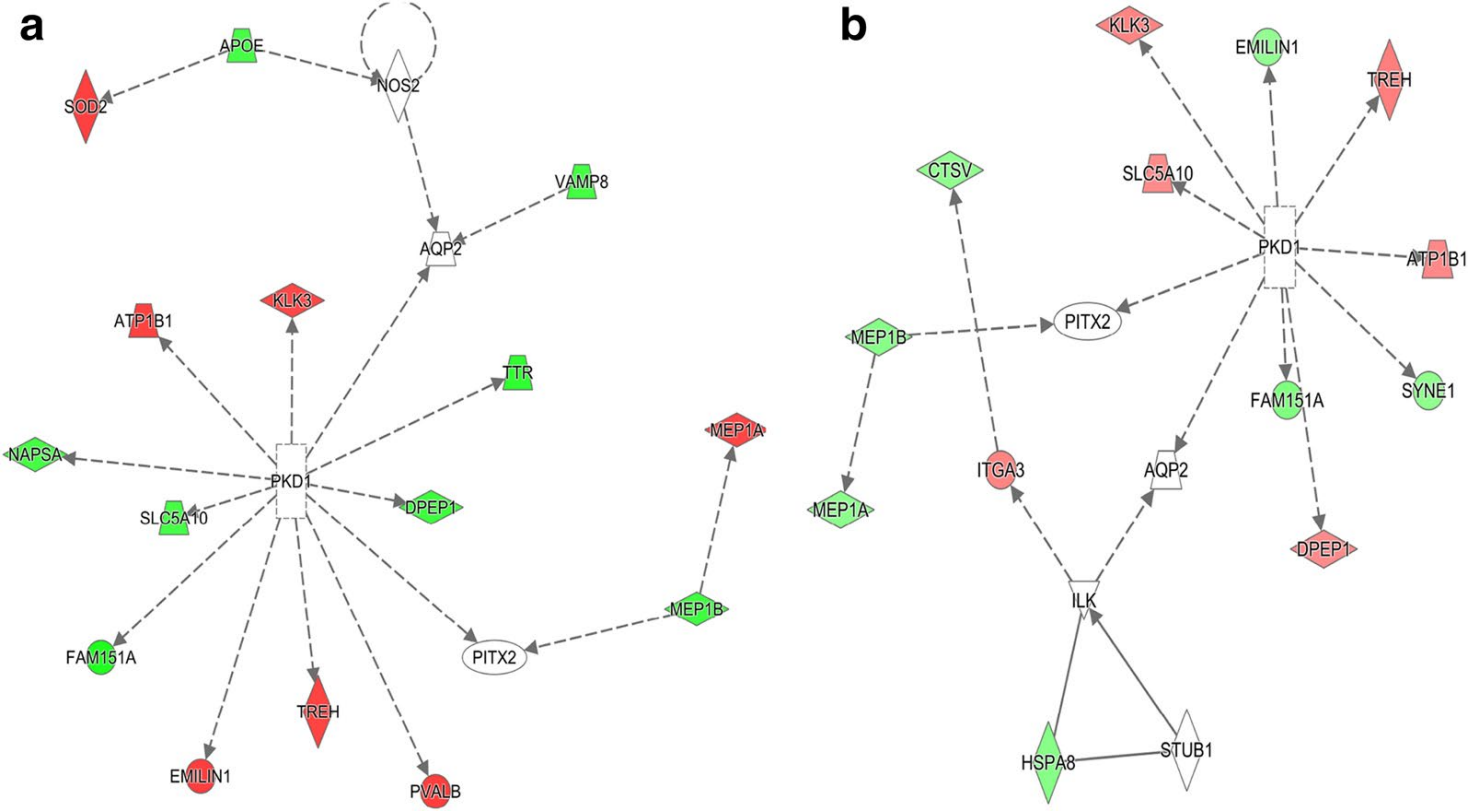
Shared top IPA network but with differences in protein regulation. **a** The IPA top-rated network for the STZ mouse model and **b** the db/db model. The fold change in the diabetic compared to the healthy control mice is illustrated with green indicating reduced levels and red indicating increased levels in the diabetic mice. In IPA, the total 505 and 395 proteins from the STZ and db/db mouse models respectively were included and several of the proteins in the top rated networks were identified in both mouse models. Of those proteins, some had opposite regulation in the two mouse models, although the difference in fold-change was too small to be significant in combination with low power due to low sample number; STZ, n = 6 in each group and db/db, n = 5 in each group

Ingenuity pathway analysis (IPA) was conducted based on the fold changes of all identified proteins from each mouse model. With a small discrepancy in the precise protein composition, the same top network was identified in the two mouse models, although some of the included proteins had oppositely regulated protein abundances (Fig. 4a, b). The four top rated identified networks within each mouse model are listed in Table 3 with the proteins included in the network and the major function of the involved proteins. The top rated network involved small molecule biochemistry and renal and urological system development. Included in the network were ATP1B1, F151A and MEP1B, all three among the VIP proteins in the separation of the diabetic and healthy mice identified in the multivariate analysis. The proteins in the subsequent networks 2, 3 and 4 were overlapping within the two mouse models. In those networks ITGB1 (Fig. 3c) was included, one of the common 27 VIP proteins with inversed protein expression in the two mouse models. Furthermore, laminin-α1 (LAMA1) appeared in the top 2–3 rated IPA networks and although not included among the VIP proteins due to different Uniprot accession numbers (P19137 in the STZ and F8VQ40 in the db/db mice, 99.7 % shared sequence similarity), it had significantly lower protein expression in the diabetic compared to the healthy mice in both mouse models (Fig. 3b).

**Table 3:** Proteins in the top rated networks in IPA in both mouse models conform

	Molecules in network	Score	Focus mol.	Top diseases and functions
*db/db*				
1	AQP2, ATP1B1, CTSV, DPEP1, EMILIN1, FAM151A, HSPA8, ILK, ITGA3, KLK3, MEP1A, MEP1B, PITX2, PKD1, SLC5A10, STUB1, SYNE1, TREH	11	13	Cell-to-cell signaling and interaction, renal and urological system development and function, organ morphology
2	AQP11, BICC1, CA3, CLCN5, CLU, CUBN, EGF, EGFR, ITGA1, ITGB1, LGMN, LRP2, SLC34A2, SOD1	6	8	Organ morphology, organismal development, renal and urological system development and function
3	ACE2, COL4A3, CTGF, FN1, LAMA1, LAMA5, LAMB1, LAMB2, LAMC1	5	6	Tissue development, decreased levels of albumin, tissue morphology
4	ADIPOQ, ATP2B1, BGN, CALB1, COL18A1, COL1A1, COL1A2, COL4A1, COL4A3, CTGF, CYP27B1, DCN, FAS, FBN1, FCGR2B, ICAM1, IFNG, ITGAV, KL, LEP, LUM, PARP1, SLC34A1, SLC9A3, SLC9A3R1, SMAD4, SMAD7, SPARC, TGFB1, TNF, TNFRSF1B, TRPV5, UMOD, VCAM1, VDR	5	11	Cellular movement, hematological system development and function, immune cell trafficking
*STZ*				
1	AK2, ALDOB, AQP1, ATP1B1, AVPR2, DPEP1, EMILIN1, FAM151A, KLK3, MEP1A, MEP1B, MIOX, NAPSA, PITX2, PKD1, SLC5A10, TREH, TTR	10	13	Small molecule biochemistry, hematological system development and function, tissue development
2	ACE2, ACTA2, AQP11, BICC1, CLU, COL1A1, COL1A2, COL4A1, COL4A3, CTGF, Ccl2, EGF, EGFR, FCGR2B, FN1, ICAM1, ITGA1, ITGAV, ITGB1, LAMA1, LAMA5, LAMB1, LAMB2, LAMC1, PARP1, PKD1, RALBP1, SMAD3, SMAD4, SMAD7, TGFB1, TNF, TNFRSF1B, UMOD, VEGFA	8	16	Organismal injury and abnormalities, cellular movement, hematological system development and function
3	BGN, COL6A3, DCN, FAS, FBN1, LUM	4	5	Hair and skin-, skeletal and muscular system-development and function, organ morphology
4	CA2, CALB1, CYP27B1, EZR, KL, MSN, S100G, SLC34A1, SLC9A3R1, TRIM24, TRPV5, VDR	3	6	Drug metabolism, lipid metabolism, molecular transport

The top rated IPA networks are shown with the included proteins together with the IPA score, the number of molecules included from the dataset and the major diseases and functions of the networks. Several of the same proteins were included in network 1 from both mouse models and although all networks are slightly overlapping, there is major overlap between networks 2–4 in both mouse models. Although the same proteins were included in the networks in both mouse models, they were not always regulated similarly

### The effect of insulin on the db/db mice

In addition to the differences and similarities between the N-glycosylated proteomes of the STZ and db/db mouse models, we investigated the effect of insulin on the db/db mouse model. The db/db insulin group was poorly separated from the db/db vehicle mice in the unsupervised PCA (Fig. 2c) although a 3D projection of the PCA showed that the db/db insulin and db/db vehicle groups were separable (see Additional file 2: Panel C). The db/db insulin group was clearly separated from the db/db vehicle in the supervised OPLS analysis (Fig. 2d). When comparing the db/db insulin mice to the db/db vehicle mice, 173 of the 395 identified proteins had a VIP score above 1 in the OPLS model and a hierarchical clustering resulted in 37 proteins with *P* < 0.05.

**Fig. 5:**
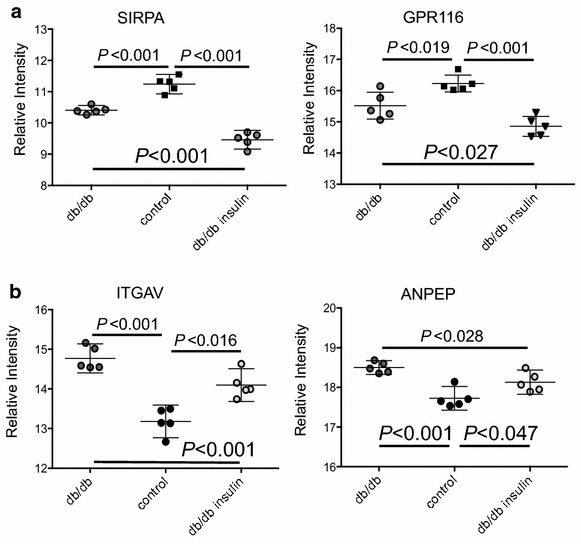
The effect of insulin on the db/db mouse model. **a** The effect of insulin on the db/db mice away from the protein levels in the healthy db/+ mice. **b** Proteins with significant regulation in the db/db mouse model where insulin had an effect on the protein abundance towards the level in the healthy db/+ mice. There was no significant regulation in the STZ mouse model. Two-tailed Student’s t test was used for calculation of significance level when 2 groups were included and one-way ANOVA with Tukey post hoc test was used for the calculations including the db/db insulin group in the db/db mouse model. Graphs are shown with mean and 95 % CI and *P* < 0.05 is considered significant

Insulin had a significant effect on the protein abundance of F151A, SLC1A6, Hyaluronoglucosaminidase-2 (HYAL2) and SIRPA (Figs. 3b, 5a), included in the VIP proteins and there was a trend towards an effect on an additional four of the 27 proteins, namely CDHR5, carbonic anhydrase-12 (CAR12), CD97 and gamma-glutamyltransferase 5 (GGT5) as shown in Table 2 and Additional file 1d. Insulin also had an effect on 12 proteins not included among the VIP proteins but within the 153 proteins common to both the STZ and db/db mouse models. These 12 proteins are listed in Table 4 with the significance of the protein expression levels, supplementary protein data can be found in Additional file 1c. In three of these proteins; LAMA1, GPR116 and activated leukocyte cell adhesion molecule (ALCAM) insulin had an effect on the protein abundances in the db/db mice away from the healthy db/+ mice (Figs. 3b, 5a). In tripeptidyl peptidase I (TPP1), low-density lipoprotein receptor-related protein-2 (LRP2) and cleft lip and palate associated transmembrane protein-1 (CLPT1) insulin increased the abundance in the db/db mice towards the levels in the healthy db/+ vehicle mice and in 6 proteins here among hypoxia-upregulated protein-1 (HYOU1), alanyl (membrane) aminopeptidase (ANPEP) and sodium channel beta-4 subunit (SCNB4) insulin decreased the abundances, also in this case towards the protein levels in the healthy db/+ control mice (example in Fig. 5b).

**Table 4:** Insulin effect on protein abundance in the db/db mouse model

Uniprot ID	Description	Short name	db/db versus db/+		db/db insulin versus db/db		Kidney compartment^B^
			Protein abundance level	*P* value	Protein abundance level	*P* value	
*Away from healthy control*							
P19137/F8VQ40^A^	Laminin subunit alpha 1	LAMA1	Down	<0.039	Down	<0.006	G = M, T = H
G5E8Q8	Adhesion G protein-coupled receptor 116	GPR116	Down	<0.019	Down	<0.027	ND, RNA T = H, G = L
E9Q3Q6	CD166 antigen, activated leukocyte cell adhesion molecule, unreviewed	ALCAM	Up	<0.018	Up	<0.033	T
*Insulin increases protein abundance towards healthy control*							
Q3TDY6	Tripeptidyl peptidase I, PUP	TPP1	Down	<0.005	Up	<0.005	G = L, T = H
A2ARV4	Low-density lipoprotein receptor-related protein 2	LRP2	Down	<0.001	Up	<0.023	T
Q8VBZ3	Cleft lip and palate associated transmembrane protein 1	CLPT1	Down	<0.001	Up	<0.01	ND, RNA T = H, G = L
*Insulin reduces protein abundance towards healthy control*							
Q9JKR6	Hypoxia up-regulated protein 1	HYOU1	Up	<0.081	Down	<0.029	ND, RNA T
Q1MXF9	Sodium channel beta4 subunit, unreviewed	SCN4B	Up	<0.001	Down	<0.001	G = L, T = M
Q3U4F3	Acid phosphatase 2, lysosomal, PUP	ACP2	Up	<0.019	Down	<0.004	ND, RNA T
A2AKI5	Integrin alpha-V light chain, unreviewed	ITGAV	Up	<0.001	Down	<0.016	G = H, T = M
Q3UP74	Alanyl (membrane) aminopeptidase, PUP	ANPEP	Up	<0.001	Down	<0.047	T
Q9JHJ8	Inducible T-cell co-stimulator ligand	ICOSL	Up	<0.001	Down	<0.007	G = L, T = M

In addition to the effect insulin had on some of the 27 VIP proteins, the abundances of 12 of the 153 proteins identified in both mouse models were affected by insulin. The protein abundance level shown as up or down and the *P* values are valid for the first group compared to the second group (db/db compared to the db/+ and db/db insulin compared to db/db respectively). Protein data is reported as in Table 2, but the STZ mouse data is not shown, as there were no significant differences except for in LAMA1. ^A^LAMA1 was identified in the STZ mouse model solely with Uniprot accession P19137 and in the db/db mouse model solely with Uniprot accession F8VQ40 (unreviewed). The sequences share 99.7 % identity and differ in length by 1 amino acid (aa) (3084 and 3083 aa respectively). The abundances of P19137 and F8VQ40 were assumed comparable. ^B^The kidney compartment reported in the last column corresponds to the findings reported in the Human Protein Atlas [28]. One-way ANOVA with Tukey post hoc test for multiple comparison correction was used for *P* value calculations, *P* < 0.05 was considered significant. *PUP* putative uncharacterized protein

## Discussion

In this study we focused on the N-linked glycosylated proteome of the kidney in two well-known mouse models for diabetes with early DN; the STZ model where diabetes is induced by repeated intermediate doses of STZ resulting in a reduction of the beta cell mass and the obese db/db model with insulin resistance and later beta cell failure [19]. In both the STZ and the db/db mice we saw increased levels of HbA1c% indicative of diabetes and increased albuminuria compared to the healthy control mice, implying early traits of DN. There was a comparable increase in HbA1c% between the healthy control and the diabetic groups in the two mouse models indicating similar degrees of diabetes despite the separate etiology in the two mouse models. Diabetes nephropathy is a late complication that in T1DM develops a decade or later after diabetes is diagnosed [29]. It is therefore noteworthy that no mouse has a lifespan that is long enough to allow them to reach the later stages of renal failure seen in human subjects with DN [19].

We detected a higher number of significant protein changes within the db/db mouse model compared to the STZ mouse model between the shared proteins. An explanation could be that some of the proteins with the largest differences within the STZ mouse model not were identified in the db/db mouse model and were not included in the model-to-model comparison. Five of these STZ mouse model specific proteins are shown in Additional file 2: Panels A–B. Proteins not detected in one mouse model could be present at lower levels in the mouse model or could have lower levels of N-glycosylation which, with the N-glycosylation capture-technique we use, would result in a lower yield of peptides. In the comparison of the two mouse models we found that most protein abundances were unchanged or similarly regulated. However, the regulation of some proteins was inverted in the db/db vehicle and STZ mice compared to the healthy control mice in the respective mouse model, with increased protein abundance in the diabetic mice in one model while decreased abundance was observed in the other. Some of the proteins with differentiated protein abundances are summarized with their possible relation to kidney damage or diabetes in Additional file 1e.

Most extracellular, secreted and cell surface proteins are N-glycosylated [30] and the majority of the proteins we identified were cell surface proteins or associated with the extracellular space. Interestingly, three of the four proteins that we identified with the inversed regulation in the STZ and db/db mouse models mainly reside in the proximal tubules in human according to The Human Protein Atlas [28]. The three mainly tubular proteins SGLT1, PROM1 and ALPL all had decreased protein abundances in the STZ and increased abundances in the db/db vehicle mice compared to the healthy control mice in the respective mouse model. The sodium/glucose co-transporters (SGLT) reabsorb glucose from the primary urine to the blood [31] and different expression pattern of SGLT1 in the two mouse models indicates that there could be DM type-specific differences in the protein abundance of this sodium glucose co-transporter. Prominin-1 is a cholesterol binding protein in the proximal tubules. No connection between DN and altered levels of PROM1 has previously been reported to our knowledge, but in a STZ mouse model of retinal vasculopathy and neuropathy, also secondary complications to diabetes, PROM1 in the photoreceptors was shown to be destroyed by metalloprotease (MMP)-9 whereas no retinopathy or neuropathy was seen in *MMP*-*9* knockout mice [32]. Those results indicate that the presence of PROM1 could be involved in reducing micro vascular damage in retinopathy since the lack of PROM1 is associated with retinopathy. Similar effects could take place in the proximal tubules in the STZ mice where PROM1 abundances were decreased compared to the NoSTZ healthy control mice. The tubular protein ALPL, also diametrically regulated in the STZ and db/db mouse models is proposed as a urinary biomarker for tubular damage in DN [33]. Tubular changes has long been known to be correlated to alterations in the glomerular filtration rate [34] and these changes could have a closer connection to the initial states of progression in DN compared to glomerular changes [35].

Several of the proteins involved in the four top-rated networks in IPA were integrin and laminin subunits. Integrins are involved in cell adhesion, cell–matrix interactions and serve as receptors for several laminin subunits [9]. Here an inverse regulation of ITGB1 (β1) protein abundance was seen in the two mouse models (Fig. 3c) whilst a similar regulation of both ITGA3 (α3) and LAMA1 abundances were observed in the mouse models [36, 37]. The changes we see in the integrin and laminin proteins indicate that there are structural changes taking place in the kidney at a very early stage of DN and some of them could be etiology specific.

Insulin is probably the most important tool used in diabetes care to control hyperglycemia and thereby to a large degree reduce the risk of late complications like DN. We saw that insulin had a clear effect on several of the renal proteins, in general tubular [28], in the db/db mice, mainly in the direction of the healthy db/+ control mice but also in the opposite direction. The level of the glomerular protein SIRPA was already decreased in the db/db vehicle mice compared to the healthy db/+ vehicle mice and insulin decreased the abundance even further. It has been shown to interact with nephrin in the slit diaphragm of the podocyte [38] and could be involved in podocyte injury [39]. Insulin also significantly decreased the abundance of GPR116 similar to SIRPA in the db/db mice (Fig. 5a) and it has been shown in mouse adipose tissue that deletion of *Gpr116* could cause impaired insulin tolerance [40]. The unchanged protein abundance of GPR116 in the STZ mouse model compared to the decreased abundances in the db/db mice indicate mouse model specific differences that could be connected to an altered body weight in the db/db mouse.

In the majority of the proteins affected by insulin, the protein levels in the db/db mice were changed towards the levels seen in the healthy db/+ mice. The effects we observe of insulin on the protein abundance levels in the db/db mice are indicative of a protective effect. However, in the cases where the protein levels are further away from the healthy db/+ mice than the levels in the db/db vehicle mice, the effect of insulin could either be detrimental or compensatory, this we do not know.

## Conclusions

We focused on ECM and cell surface kidney proteomics using N-linked glycosylated peptide enrichment and showed that there were significant protein differences between the STZ and db/db mouse models. Our data suggests that DM type specific protein differences in the kidney could precede later shared morphological alterations in DN.

We also show that insulin changes the N-glycosylated protein abundances in the db/db mouse model both in the direction of the levels in the healthy control mice and in the opposite direction. These insulin induced changes in protein abundance in the db/db mouse kidney could be adipose tissue related, caused by reduced hyperglycemia or other systemic effects of insulin in the mice, by insulin signaling in the kidney or could possibly compensate for the adverse effects of diabetes in the animals. Insulin administration, crucial for survival in type 1 DM but to a large extent replaceable in type 2 DM, has a distinct effect on the N-glycosylated proteome in the db/db mice that could be of high interest to compare with the effect of other type 2 DM intervention strategies.

## Methods

### Animals

The handling and use of all animals in the present study was approved by The Danish Animal Experiments Inspectorate and was carried out according to the guidelines of “The Council of Europe Convention for the protection of vertebrate animals used for experimental and other scientific purposes”.

Male 129SV mice 6 weeks of age and male db/db and db/+ mice 8 weeks of age were purchased from Charles River, Germany and fed Altromin 1324 and tap water ad libitum from arrival. The animals were housed in the animal unit facility at Novo Nordisk, Denmark (SV129 mice 10/cage and db/db and db/+ mice 5/cage) in controlled temperature (19–21 °C for SV129 and 23–24 °C for db/db) and a 12 h light, 12 h dark cycle. After 2 weeks of acclimatization one group of randomly selected 129SV mice were given STZ injections intraperitoneally twice at a dose of 125 mg/kg with 3 days between doses. Two weeks post STZ intervention, BG measurements were conducted and mice in the STZ group (STZ) with elevated BG above 16 mM were included in the experiment together with the group of SV129 mice that was not given STZ (NoSTZ). Concomitantly vehicle dosing (s.c. 4 mL/kg QD, vehicle composition: pH 7.4; 20 mM phosphate; 130 mM sodium chloride; 0.05 % polysorbate 80) was initiated of the STZ induced diabetic and healthy NoSTZ groups of mice.

After 2 weeks of acclimatization of the db/+ and db/db mice, BG measurements were conducted and db/db mice with BG above 16 mM were included in the experiment. The db/db mice were divided into dosing groups given vehicle (db/db vehicle) or insulin (db/db insulin) while the healthy db/+ control mice (db/+) were given vehicle. Vehicle composition and dosing was as with the STZ mice except for dosing volume (2 mL/kg) due to the larger size of the db/db mice. The first dose of insulin was 2 LinBits per 20 g mouse plus 1 LinBit (LinShin Inc, Scarborough, ON, Canada) for each additional 5 g of mouse. The insulin dose was thereafter adjusted according to BG profiles with insulin glargine (Nomeco A/S, Copenhagen, Denmark) at a dose of 10 U/kg twice a day. During the study the animals were weighed once weekly on a digital scale.

### *In vivo* measurements

Blood glucose was analyzed once weekly on a Biosen S-line/5040 (EKF-diagnostics, Magdeburg, Germany) and HbA1c% was analyzed on a Cobas 6000 autoanalyzer (Roche Diagnostics Ltd, Rotkreuz, Switzerland) once weekly for the db/db mice and at 6 and 10 weeks after STZ dosing for the STZ mice. Blood was taken at all time points from non-fasted mice before dosing.

Metabolic cages (Techniplast S.p.A., Buguggiate, Italy) for individual collection of urine was used at baseline, 6 and 12 weeks (age 10, 16 and 22 weeks) for the db/db mice and at 2, 6 and 10 weeks (age 10, 14 and 18 weeks) after the STZ intervention for the STZ mice. Urine albumin excretion determination (AER) was determined using a sandwich ELISA (Bethyl Labs, Montgomery, TX, USA).

The animals in the db/db mouse model were sacrificed after 12.5 weeks of insulin dosing and the animals in the STZ mouse model were sacrificed 10 weeks after the STZ intervention by perfusion under isoflurane anesthesia with 20 mL 0.9 % NaCl with heparin (10 U/mL). Kidneys were weighed individually after having the surrounding fat removed and were snap frozen in liquid nitrogen. The left kidney from the db/db and the right kidney from the STZ animals were studied.

In the STZ mouse model, the total number of analyzed mice in the proteomics study was n = 12 with n = 6 STZ induced diabetic SV129 and n = 6 NoSTZ SV129 healthy controls, both groups dosed with vehicle. The total number of analyzed animals in the db/db mouse model part of the study were n = 15 with n = 5 db/db diabetic vehicle dosed, n = 5 db/db diabetic insulin dosed and n = 5 healthy db/+ control vehicle dosed mice (Fig. 1a, b).

### Protein purification

Snap frozen kidney tissue was transferred to a Denator Stabilizor T1 [41] (Denator, Gothenburg, Sweden). From the animals in the STZ mouse model, 50 mg of tissue was used resulting in one sample/animal and for the animals in the db/db mouse model 100 mg of tissue, divided into two technical replicates, were used. The purification was done as described in Kurbasic et al. [13] based on the original paper by Zhang et al. [18]. The protein concentration was determined using the Micro Lowry assay kit Peterson’s Modification (Sigma-Aldrich, Stockholm, Sweden). In brief, the homogenized tissue was degraded with 10 μg/mL sequencing grade modified Trypsin (Promega, Madison, WI, USA) oxidized with sodium periodate (Serva, Heidelberg, Germany) to a final concentration of 8 mM and coupled to hydrazide Affi-gel (Bio-Rad, Hercules, CA, USA). Unbound peptides were washed off the hydrazide Affi-gel, where after the N-glycosylated peptides were cleaved off overnight by 5 U (1 U/μL) PNGase F (Roche Gmbh, Mannheim, Germany), dried on a SpeedVac (Thermo Scientific, Waltham, MA, USA), cleaned up by reverse-phase C18 chromatography (Waters, Milford, MA, USA) and dried again.

### Mass spectrometry

All solvents for high performance liquid chromatography (HPLC) were from Sigma-Aldrich and percentages are reported as (v/v). Samples were dissolved in 5 % acetonitrile (ACN) and 0.1 % formic acid (FA) for analysis on an linear trap quadropole (LTQ) Orbitrap XL mass spectrometer (Thermo Electron, Bremen, Germany). Peptide separation was carried out using an Eksigent 2D NanoLC system (Eksigent Technologies, Dublin, CA, USA) where the mobile phase A was water/0.1 % FA and the mobile phase B was ACN/0.1 % FA. Loading, washing and separation was performed as in Kurbasic et al. [13]. Peptides were eluted from the analytical column using a linear gradient of mobile phase B developed from 3–35 % B during 60 min. The gradient was followed by 20 min column washing with 90 % ACN, 0.1 % FA and a 15 min re-equilibration with 3 % B. Peptides were analyzed using data-dependent acquisition, simultaneously scanning a mass range between 400 and 2000 Da in the Orbitrap and MS/MS spectra in the LTQ. Four MS/MS spectra were collected per second using collision-induced dissociation (CID) in the LTQ ion trap. The normalized collision energy was set to 35 %. Each Orbitrap MS scan was acquired at 60000 FWHM nominal resolution settings using the lock as mass option (m/z 445.120025) for internal calibration. A dynamic exclusion list restricted to 500 m/z values was used for 2 min with a repeat count of 2. Data was acquired using Xcalibur software, version 2.0.7 (Thermo Fischer Scientific, Hägersten, Sweden).

### Data processing

Each mouse model was examined as an individual project and all data comparisons were made within the project that the samples belonged to. Raw mass spectrometric data were analyzed with the in-house developed software Proteios SE (version 2.19.0) [42] and also independently with Progenesis QI v. 1.0.5156.29278 (Nonlinear Dynamics, Waters). In Progenesis, raw data was automatically aligned, manually inspected and adjusted. There is a built-in quality assessment in Progenesis where a 3-step color code for the spectrum alignment is used (green = good, beige = ok and red = needs revision). For both the STZ and db/db datasets, alignments were in good or ok agreement (green or beige). All detected features were exported to Mascot (MatrixScience, London, UK) version 2.4.1 and searched against the UniprotKB mouse 2015.08 database with equal number of reversed sequences. The parent ion mass tolerance was set to 5 ppm and to 0.8 Da for the fragment ions and the MS/MS ion charge was set to 2+/3+/4+. One missed protease cleavage was allowed. Cys carbamidomethylation was set as fixed modification and Met oxidation and Asn to Asp deamidation as variable modification (gain of 0.984 Da) as a consequence of PNGase F cleavage. Peptide identifications were propagated between runs and both peptides and proteins were filtered at 5 % FDR. Peptides with reverse sequences and no Asn to Asp modification were removed. Only proteins with a Mascot score > 25 and at least one unique peptide were kept. Protein grouping was employed in Progenesis. No missing value imputation was done. The results from Proteios and Progenesis were comparable.

The mass spectrometry data have been deposited to the ProteomeXchange Consortium [43, 44] via the PRIDE partner repository with the dataset identifier PXD003196 and 10.6019/PXD003196 for the STZ project and PXD003349 and 10.6019/PXD003349 for the db/db project. A guide to the file names is listed in an Additional file 1f. After peptide identification and protein assembly in Progenesis, the processed non-normalized data intensities from both mouse models were run through the software Normalyzer 1.1.1 [45, 46]. Normalyzer assesses the optimal normalization method for omics data sets compared to log2 transformation and the data sets are validated from both quantitative and qualitative perspectives, since the optimal normalization method is dependent on the intrinsic characteristics of the data set [45, 46]. Both the STZ and db/db data sets were normalized using Loess-G [47]. Mean values were used for the statistical analysis of technical replicates. Of the 153 proteins identified in both mouse models, peptides with the highest scores were manually examined for the NXS/T motif.

### Multivariate data analysis

Unsupervised principal component analysis (PCA) and supervised orthogonal partial least square discriminant analysis (OPLS-DA) were conducted in SIMCA v. 14 (Umetrics, Umeå, Sweden) with univariate scaling. Both analysis methods reduce the dimensionality in the dataset by introducing components that describe the joint behavior of several variables. The major difference between the unsupervised and the supervised methods is that in the PCA the first component is the one describing the largest co-variance within the dataset, regardless of direction [48] and in the OPLS-DA, the first component is class dependent and describes the major difference between the included groups, separating them on the x-axis [49]. In the PCA, the second component is composed of the variables with the largest orthogonal co-variance to the first one [48]. In the OPLS-DA analysis, the orthogonal difference takes place on the y-axis, identifying differences within the groups [49, 50]. The PCA and OPLS model statistics R2X(cum), R2Y(cum), Q2(cum) [51] and PCA components are summarized in an Additional file 2: Panel A.

In this study, OPLS-DA was used to compare the N-glycosylated proteomes of the diabetic to the healthy mice within the STZ and db/db mouse models and to compare the db/db insulin to the db/db vehicle group. The most important protein variables for the separation of the mouse groups in the OPLS-DA can be seen in the Variable Importance for the Projection (VIP)-plot. Values above 1 indicate importance for the OPLS model. The VIP plot of the STZ mouse model was compared to the VIP plot of the db/db mouse model not including the db/db insulin group. Proteins identified both in the STZ and db/db mice with a VIP score above 1 were used in the comparison of the two mouse models. Proteins separating the db/db insulin from the db/db vehicle group were also examined. An overview of all the models is shown in Additional file 2: Panel A.

Hierarchical clustering of both the variables and mouse samples was done in Qlucore v. 3.2 (Qlucore AB, Lund, Sweden) where the cut-off level was set to *P* < 0.05. Proteins with significant expression were evaluated using univariate analyses.

### Statistics for univariate data

For univariate comparisons between the two groups in the STZ mouse model, two-tailed Student’s t test for equal standard deviations (SD) was performed in GraphPad Prism 6 (La Jolla, CA, USA), which simultaneously calculates the F-test for equal SD. For multiple group comparisons within the db/db mouse model one-way ANOVA and Tukey post hoc test was used simultaneously with Brown-Forsythe (BF) test to compare intra group variance. In all tests *P* < 0.05 was considered significant. In the text, mouse parameters and protein data are presented as mean ± SD and in the graphs mouse parameters are presented with mean ± standard error of mean (SEM) and protein data with means and 95 % confidence intervals (CI). Peptide data for the 153 shared proteins are listed in Additional files 1a–b and protein mean intensities and SD for the proteins listed in Tables 2, 4 and Additional file 1d are reported in Additional file 1c. All calculations were done in GraphPad PRISM 6, and AmberBio (Amber BioScience, Lund, Sweden).

### Biological relationships

Protein network analysis was performed with Ingenuity Pathway Analysis (IPA) (Ingenuity Systems, Mountain View, CA, USA). In IPA, individual protein relationships obtained from the literature are gathered into hypothetical interaction networks [52, 53]. The MS obtained normalized intensities were converted to fold change for all proteins comparing the diabetic to the healthy control mice. The two mouse models were entered separately into IPA and core analysis was performed for kidney tissue in mice, allowing the maximum default of 35 proteins per network. Scores of 2 or above have 99 % confidence or more of not being generated by chance. The acquired top rated networks and canonical pathways were compared between the two mouse models.

## Additional files

10.1186/s12014-016-9123-z Additional file 1 includes additional excel sheets 1a-f where 1a shows shotgun peptide data from the STZ mouse model, formerly N-glycosylated peptides for the 153 common proteins, 1b shows shotgun peptide data from the db/db mouse model, formerly N-glycosylated peptides for the common 153 proteins, 1c shows the relative mean intensities, SD, Mascot score, peptide count and number of unique peptides used in the identification of the shared proteins of importance in the multivariate analyses and the proteins where insulin had an effect on the abundances. 1d shows proteins identified in both mouse models with significant protein abundances in the db/db mouse model but not in the STZ mouse model, 1e show protein characteristics in relation to diabetes or kidney damage for a selection of the proteins with significantly changed protein abundances and 1f shows a conversion table of Proteome Exchange filenames and the corresponding kidney sample name and mouse group.

10.1186/s12014-016-9123-z Additional file 2 includes panels A-C where panel A shows a table of the SIMCA statistics describing the fit of the PCA and OPLS-DA analyses for the STZ and db/db mouse models. Values are between 0 and 1, where 1 is the perfect fit. R2X(cum) reflect the fraction of variation in X explained by the model, R2Y(cum) reflect the fraction of variation in Y explained by the model and Q2(cum) reflect the fraction of variation predicted by the model. A is the number of components in the model. In the PCA, the components are shown as R2X[n]. Panel B shows a turned 3D PCA illustration of the STZ mouse model, revealing that there is a degree of separation between the STZ and NoSTZ groups. The first principal component does not separate the mouse groups. Panel C shows a turned 3D PCA illustration of the db/db insulin and db/db vehicle groups.

10.1186/s12014-016-9123-z Additional file 3 includes panels A and B and shows proteins only identified in the STZ mouse model with significant protein abundances. Panel A shows graphs of selected proteins significance is calculated with two-tailed Student’s *t* test for equal variance, *P* < 0.05 is considered significant. Mean and 95 % CI are shown in the graphs. Panel B shows a summary table for the same proteins with q-values, Mascot score, peptide count and number of unique peptides used in the quantification.

## Abbreviations

AER: albumin excretion rate
BG: blood glucose
BM: basement membrane
db/db: leptin receptor deficient
DN: diabetic nephropathy
ECM: extracellular matrix
ER: endoplasmic reticulum
HbA1c%: glycated hemoglobin A1c
LC-MS/MS: liquid chromatography tandem mass spectrometry
OPLS-DA: orthogonal partial least square discriminant analysis
PCA: principle component analysis
STZ: streptozotocin
T1DM/T2DM: type 1 or 2 diabetes mellitus
