# Supplementary material for: N-glycosylation proteome enrichment analysis in kidney reveals differences between diabetic mouse models
Source: Clin Proteomics. 2016 Oct 15;13:22. doi: 10.1186/s12014-016-9123-z (PMC5065702; doi:10.1186/s12014-016-9123-z)

A

Model of	Type	R2X(cum)	R2Y(cum)	Q2(cum)	Scaling	A	No. of Samples	PCA component conribution		
STZ mouse model								R2X[1]	R2X[2]	R2X[3]
STZ vehicle - NoSTZ vehicle	PCA-X	0.577	-	0.0152	UV	3	12	0.238	0.18	0.159
STZ vehicle - NoSTZ vehicle	OPLS-DA	0.449	0.983	0.821	UV	1+2+0	12	-	-	-
db/db mouse model								R2X[1]	R2X[2]	
db/db model all three samples	PCA-X	0.712	-	0.444	UV	3	15	0.363	0.184	-
db/db vehicle- db/+ vehicle	PCA-X	0.631	-	0.330	UV	2	10	0.479	0.152	-
db/db vehicle- db/db insulin	PCA-X	0.598	-	0.228	UV	2	10	0.309	0.288	-
db/+ vehicle- db/db insulin	PCA-X	0.683	-	0.460	UV	2	10	0.439	0.244	-
db/db model all three samples	OPLS-DA	0.607	0.944	0.837	UV	2+1+0	15	-	-	-
db/db vehicle- db/+ vehicle	OPLS-DA	0.595	0.997	0.975	UV	1+1+0	10	-	-	-
db/db vehicle- db/db insulin	OPLS-DA	0.417	0.933	0.594	UV	1+1+0	10	-	-	-
db/+ vehicle - db/db insulin	OPLS-DA	0.592	0.995	0.957	UV	1+1+0	10	-	-	-

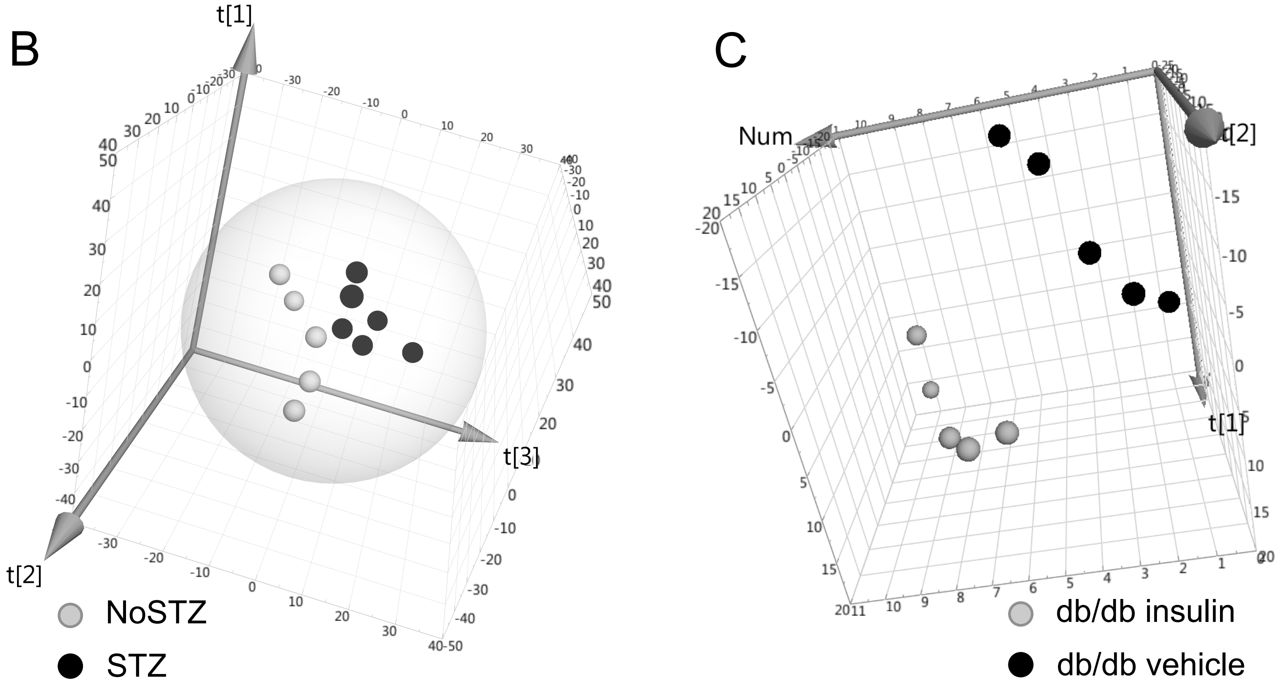

Supplement: Supplementary file 2 — 10.1186/s12014-016-9123-z Additional file 2 includes panels A-C where panel A shows a table of the SIMCA statistics describing the fit of the PCA and OPLS-DA analyses for the STZ and db/db mouse models. Values are between 0 and 1, where 1 is the perfect fit. R2X(cum) reflect the fraction of variation in X explained by the model, R2Y(cum) reflect the fraction of variation in Y explained by the model and Q2(cum) reflect the fraction of variation predicted by the model. A is the number of components in the model. In the PCA, the components are shown as R2X[n]. Panel B shows a turned 3D PCA illustration of the STZ mouse model, revealing that there is a degree of separation between the STZ and NoSTZ groups. The first principal component does not separate the mouse groups. Panel C shows a turned 3D PCA illustration of the db/db insulin and db/db vehicle groups. [file 12014_2016_9123_MOESM2_ESM.pdf]
