# Supplementary material for: N-glycosylation proteome enrichment analysis in kidney reveals differences between diabetic mouse models
Source: Clin Proteomics. 2016 Oct 15;13:22. doi: 10.1186/s12014-016-9123-z (PMC5065702; doi:10.1186/s12014-016-9123-z)
Supplement: Supplementary file 3 — 10.1186/s12014-016-9123-z Additional file 3 includes panels A and B and shows proteins only identified in the STZ mouse model with significant protein abundances. Panel A shows graphs of selected proteins significance is calculated with two-tailed Student’s t test for equal variance, P < 0.05 is considered significant. Mean and 95 % CI are shown in the graphs. Panel B shows a summary table for the same proteins with q-values, Mascot score, peptide count and number of unique peptides used in the quantification. [file 12014_2016_9123_MOESM3_ESM.pdf]

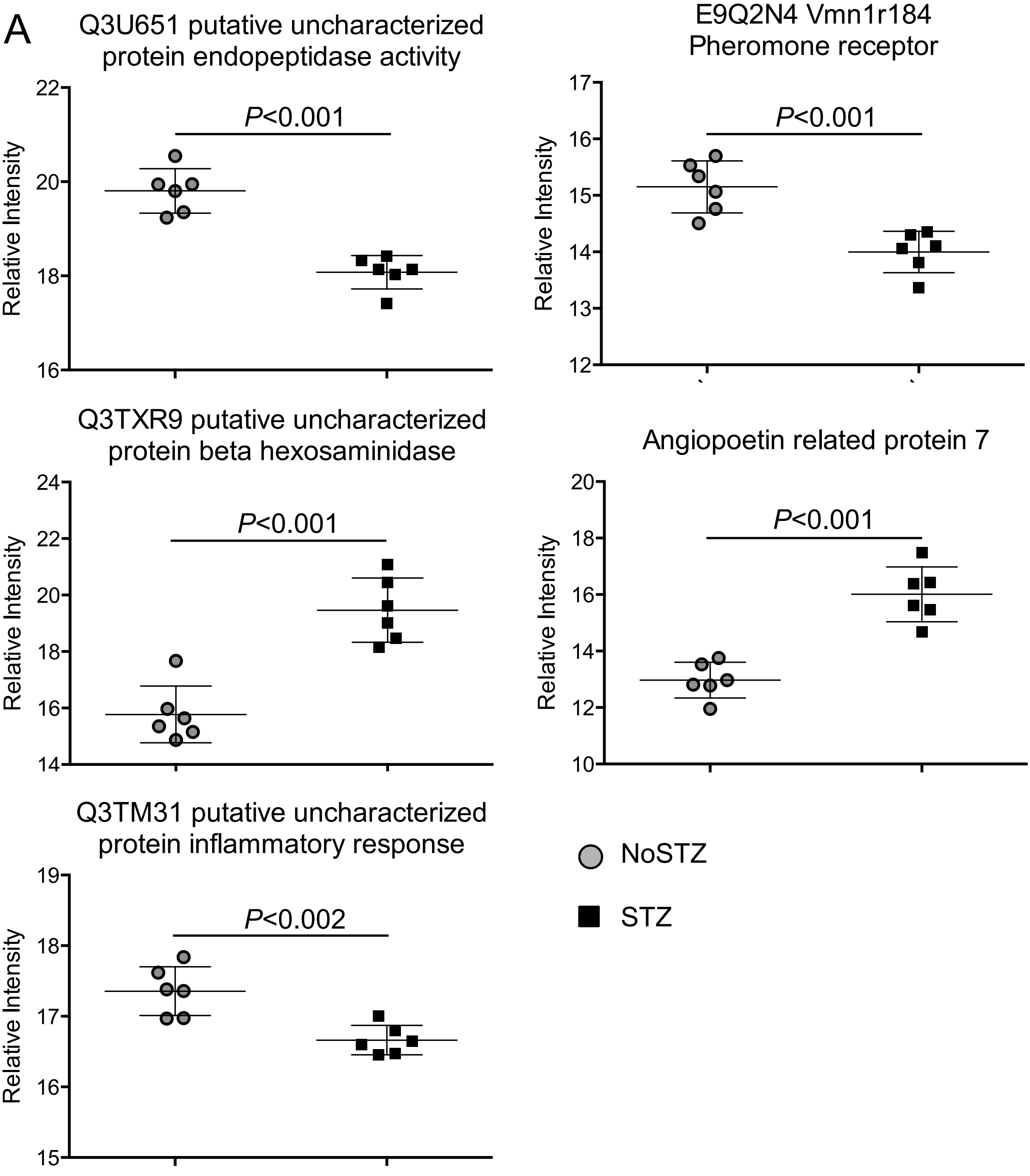

B

Uniprot accession number	Description	Short name/ Gene	STZ vs. NoSTZ	STZ t-test *P*-value	STZ q-value	Mascot score	Peptide count	Unique peptides
Q3U651	Putative uncharacterized protein, endopeptidase	Ctsd	up	<0.001	<0.007	224.63	4	3
Q3TXR9	Putative uncharacterized protein, beta hexosamine	Hexb	down	<0.001	<0.012	133.85	3	3
Q8R1Q3	Angiopoietin-related protein 7	ANGL7	down	<0.001	<0.008	170.02	3	3
E9Q2N4	Protein Vmn1r184, pheromone receptor	Vmn1r184	up	<0.001	<0.053	47.03	3	1
Q3TM31	Putative uncharacterized protein, inflammatory response	Tlr3	up	<0.002	<0.101	201.41	7	7
